# Transforming growth factor β regulates β-catenin expression in lung fibroblast through NF-κB dependent pathway

**DOI:** 10.3892/ijmm.2014.1916

**Published:** 2014-08-27

**Authors:** JIAN LI, GANG WANG, XIA SUN

**Affiliations:** 1 Jinan Centre for Disease Control and Prevention, Jinan, Shandong 250001, P.R. China; 2 Jiangsu Key Laboratory of Biological Cancer Therapy, Xuzhou Medical College, Xuzhou, Jiangsu 223002, P.R. China; 3 Department of Pediatrics, The University of Chicago, Chicago, IL 60637, USA

**Keywords:** β-catenin, LPA, TGF-β, NF-κB, pulmonary fibrosis, fibroblast

## Abstract

β-catenin contributes to the pathogenesis of lung fibrosis. However, the expression of β-catenin in fibroblasts under fibrotic conditions has not been studied. We investigated the expression of β-catenin in lung fibroblasts from bleomycin (BLM)-challenged mice and human lung fibroblasts treated with transforming growth factor β (TGF-β) or lysophosphatidic acid (LPA) by western blot analysis. The result showed that the expression of β-catenin was significantly increased in lung fibrotic foci and lung fibroblasts from bleomycin-challenged mice. TGF-β stimulated β-catenin expression and induced differentiation in human lung fibroblasts *in vitro*. Pretreatment of the NF-κB activation inhibitor attenuated the TGF-β-induced expression of β-catenin and differentiation in human lung fibroblasts. Similarly, LPA induced β-catenin expression in human lung fibroblasts, and pre-treatment of the neutralized anti-TGF-β antibody attenuated the LPA-induced expression of β-catenin and differentiation in human lung fibroblasts. The results suggested that β-catenin expression is upregulated in lung fibroblast during differentiation, and that TGF-β induced β-catenin expression in human lung fibroblasts through the activation of NF-κB.

## Introduction

Idiopathic pulmonary fibrosis (IPF) is a chronic and progressive interstitial lung disease characterized by distorted lung architecture and loss of respiratory function (1,2). Over five million individuals are afflicted with IPF in the USA, and the average survival time of IPF patients is only 2–5 years after initial diagnosis (3,4). Despite advances in understanding of the basic molecular pathways that drive this uncontrolled fibrotic process, lung transplantation remains the only effective cure for IPF (5). Thus, investigations to improve understanding of the pathological mechanisms and lead to the development of efficacious therapeutic approaches for IPF are necessary.

Fibroblast accumulation leads to excessive scarring of lung tissue, and progressive and irreversible destruction of lung architecture, leading to loss of lung function, disruption of gas exchange and fibrogenesis in lung (6). Investigations thus far have demonstrated that transforming growth factor-β (TGF-β), sphingosine 1 phospate (S1P) and lysophosphatidic acid (LPA) are involved in fibrogenesis in different organs, such as heart, liver and lung (4,6–9). Findings of recent studies suggest the crosstalk between TGF-β pathways and LPA or S1P pathways in lung fibroblasts (4,9). LPA also increases TGF-β expression and secretion in lung fibroblasts through the activation of its receptors (9). Additionally, *in vitro* investigations have shown that S1P induced the activation, differentiation and migration of lung fibroblasts (4). However, the crosstalk between the TGF-β pathway and other pathways on the effects in fibroblast differentiation remains to be determined.

Wnt/β-catenin signaling is a key regulator in tissue repair, fibrosis, remodeling or destruction (2,10,11). Immunohistochemical staining shows the increase of nuclear β-catenin staining in IPF tissue sections (12,13). Unbiased microarray screens have revealed an increased expression of Wnt/β-catenin target genes in lung tissue from IPF patients (12). Additionally, aberrant expression of extracellular matrix (ECM) proteins through epithelial-mesenchymal transition (EMT) and fibroblast differentiation contributes to pulmonary fibrosis (14–16). Mounting evidence suggest that the Wnt/β-catenin and TGF-β pathways contribute to the epithelial-mesenchymal transition (EMT) during pulmonary fibrogenesis (10,11,17,18). *In vitro*, upregulation of Wnt/β-catenin signaling promotes the proliferation of alveolar type II (AT2) cells and inhibits their ability to differentiate into alveolar type I (AT1 cells) cells (19). In lung fibroblasts, activation of Wnt/β-catenin signaling also stimulates ECM gene expression (20). However, the expression of β-catenin in lung fibroblasts has not been studied and the potential mechanism remains to be determined.

In the present study, we investigated β-catenin expression under fibrotic conditions by western blot analysis. The results showed that β-catenin expression was markedly increased in lung tissue and fibroblasts from bleomycin-challenged mice, and regulated by the activation of NF-κB.

## Materials and methods

### Reagents and kits

Bleomycin sulfate was obtained from Hospira Inc. (Lake Forest, IL, USA), and neutralizing chicken anti-TGF-β1 antibody and control chicken IgG were obtained from R&D Systems (Minneapolis, MN, USA). Oleoyl lysophosphatidic acid (18:1 LPA) was obtained from Avanti Polar Lipids (Alabaster, AL, USA), and the cell lysis buffer was purchased from Cell Signaling Technology, Inc. (Danvers, MA, USA). Protease inhibitor cocktail tablets (EDTA-free Complete) were purchased from Roche Diagnostics (Indianapolis, IN, USA). Recombinant human TGF-β1 was obtained from Protech, Inc. (Rocky Hill, NJ, USA). Horseradish peroxidase-conjugated anti-mouse IgG and anti-rabbit IgG antibodies were obtained from Bio-Rad Laboratories, Inc. (Hercules, CA, USA). Rabbit anti-fibronectin and anti-β-catenin antibodies were purchased from Santa Cruz Biotechnology, Inc. (Santa Cruz, CA, USA). Mouse anti-α-smooth muscle actin (α-SMA) and anti-β actin antibodies and Bay 11-7082 (NF-κB inhibitor) were purchased from Sigma-Aldrich (St. Louis, MO, USA).

### Experimental pulmonary fibrosis model

The animal experiment of pulmonary fibrosis was designed as previously described (4,9). Briefly, C57/BL6 mice (male, aged 8 weeks) purchased from Jackson Laboratory (Bar Harbor, ME, USA) were used for bleomycin-induced fibrosis. Briefly, C57/BL6 mice were anesthetized (with a 3 ml/kg mixture of 25 mg/kg of ketamine in 2.5 ml of xylazine), followed by treatment with saline or bleomycin sulfate (1.5 U/kg of body weight, ~0.03 units/animal) in saline by an intratracheal injection in a total volume of 50 μl. Twenty-one days post-bleomycin administration, the animals were sacrificed by cervical dislocation and the lungs were removed for histological staining and isolation of lung fibroblasts.

### Immunohistochemical staining and Masson’s trichrome staining of mouse lung tissue

Lung tissues from mice with or without bleomycin challenge were embedded in paraffin and cut as 5-μm sections for staining. Following the removal of paraffin with xylene and clearing with alcohol, the slides were applied for immunohistochemical staining and Masson’s trichrome staining and examined as previously described (9).

### Cell culture

Murine lung fibroblasts were isolated from mice with or without bleomycin (BLM) challenge as previously described (9,21). A human lung fibroblasts cell line (WI-38) was obtained from the American Type Culture Collection (ATCC; Manassas, VA, USA) and cells were grown and maintained in 6-well dishes with Dulbecco’s modified Eagle’s medium (DMEM) containing 10% fetal bovine serum (FBS). Primary murine lung fibroblasts, isolated from C57/BL6 mice with or without bleomycin treatment, were also cultured in DMEM containing 10% FBS.

### Treatment of neutralizing antibodies or NF-κB inhibitor

Serum-starved (for 24 h) human lung fibroblasts (WI-38, ~90% confluence) were pretreated with neutralized anti-TGF-β antibody or control IgG antibody (5 μg/ml, 1 h). For nuclear factor κ-light-chain-enhancer of activated B cells (NF-κB) inhibitor (Bay 11-7082), the compound was pretreated with a final concentration of 10 μM for 1 h (22). The cells were subsequently challenged with 18:1 LPA (10 μM) or TGF-β (5 ng/ml) for 48 h, and cell lysates (20 μg protein) were subjected to SDS-PAGE and western blotting.

### SDS-PAGE and western blotting

SDS-PAGE and western blotting were performed as previously described (9). The integrated density of pixels in each membrane was quantified using ImageQuant 5.2 software (Molecular Dynamics, Sunnyvale, CA, USA).

### Immunofluorescence staining

Immunofluorescence microscopy to determine protein expression was performed as previously described (9). Briefly, primary murine lung fibroblasts were grown on slide chambers for 24 h. The cells were fixed, incubated with primary antibodies (1:200 dilutions in blocking buffer) for 1 h and with Alexa Fluor secondary antibodies (1:200 dilutions in blocking buffer) for another 1 h, followed by mounting. The cells were then examined under a Nikon Eclipse TE 2000-S fluorescence microscope with a 60× oil immersion objective lens.

### Statistical analysis

Data are expressed as means ± SEM from at least three independent sets of experiments. Results were subjected to statistical analysis using one-way ANOVA or a two-tailed Student’s t-test. P<0.05 was considered to indicate a significant result (23,24).

## Results

### Expression of β-catenin in lung fibrotic foci from mice with bleomycin challenge

**Figure 1:**
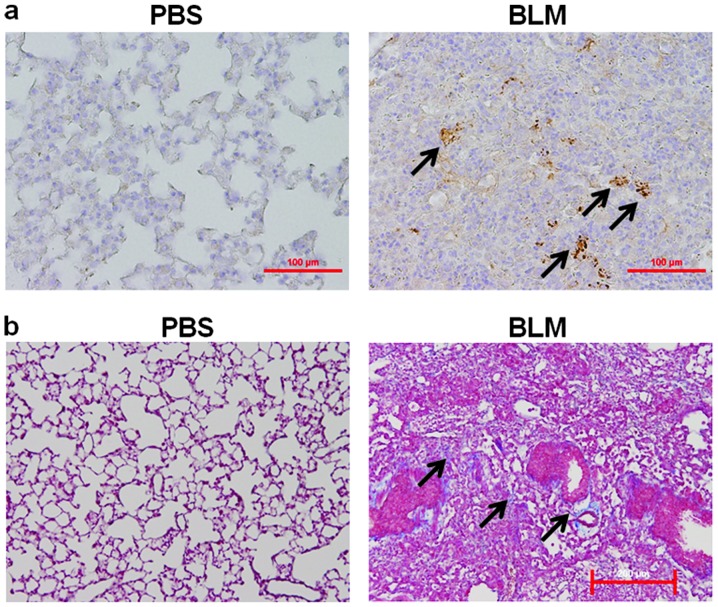
Staining of β-catenin and collagen in lung tissue from mice with or without BLM challenge. Representative immunohistochemical staining for (a) β-catenin and (b) Masson’s trichrome staining for collagen in murine lung tissue isolated from mice with or without BLM challenge. Arrow shows the marked expression of (a) β-catenin (brown color) and (b) the deposition of collagen (blue color) in fibrotic foci. Scale bar, 200 μm.

The expression of β-catenin in lung fibrotic foci from bleomycin-challenged mice was assessed. Immunohistochemical staining revealed that the expression of β-catenin was significantly increased in lung fibrotic foci from bleomycin-challenged mice (Fig. 1a). Trichrome staining revealed that the expression of collagen, bio-markers of pulmonary fibrosis, was markedly increased in these mice (Fig. 1b).

### Expression of β-catenin in lung fibroblasts from mice with or without bleomycin challenge

**Figure 2:**
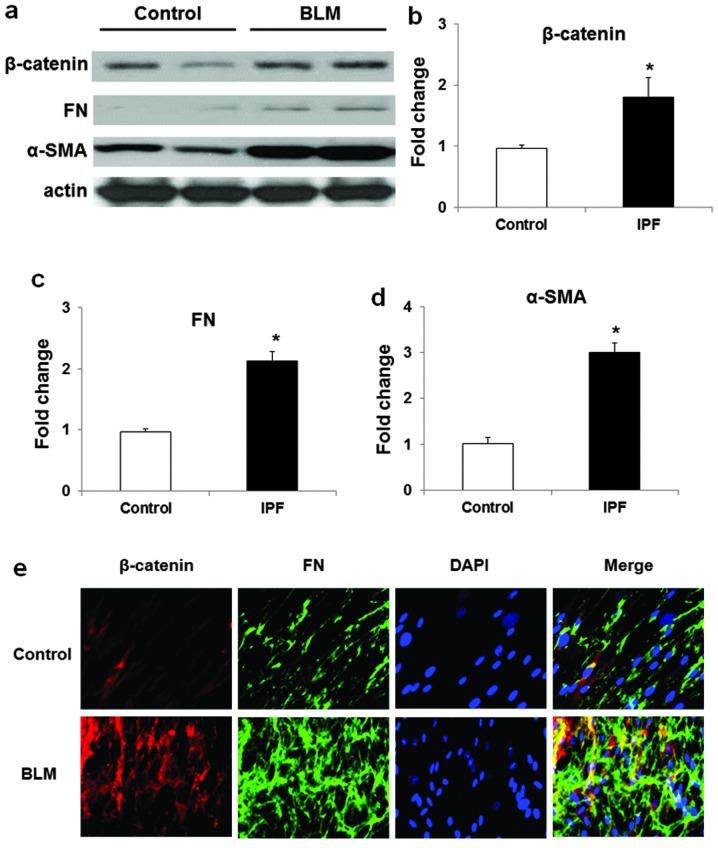
Expression of β-catenin, α-SMA and FN in lung fibroblasts from mice with or without BLM challenge. (a) Representative western blot analysis (b–d) quantification of the expression of (b) β-catenin, (c) FN and (d) α-SMA) in lung fibroblasts from mice with or without BLM challenge. Data are expressed as means ± SEM of three independent experiments. ^*^P<0.05 vs. cells from control mice. (e) Immunofloresent staining of β-catenin (red) and FN (green) in lung fibroblasts from mice with or without BLM challenge. Images were examined by immunofluorescence microscopy and recorded by using a 60× oil objective.

The expression of β-catenin in fibroblasts from control and fibrotic lungs was also examined. As shown in Fig. 2a–d, the expression of β-catenin, as well as α-SMA and FN, bio-markers of fibroblast differentiation, were markedly increased in lung fibroblasts from bleomycin-challenged mice as compared to that from the control mice. Immunofluorescence staining also showed that the expression of β-catenin was markedly increased in lung fibroblasts from bleomycin-challenged mice as compared to that from the control mice (Fig. 2e). These findings suggested that β-catenin expressed in lung fibroblasts may correlate to fibroblast differentiation under fibrotic conditions.

### TGF-β induces β-catenin expression in human lung fibroblasts

**Figure 3:**
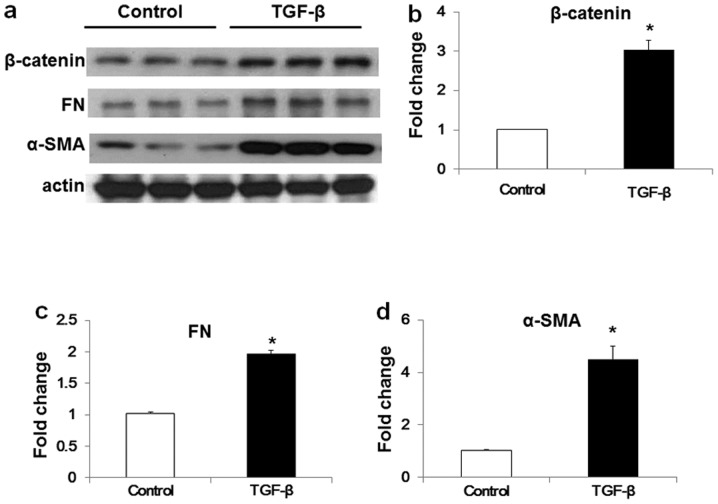
TGF-β induced the expression of β-catenin, FN and α-SMA in human lung fibroblasts. (a) Representative western blot and (b–d) quantification of the expression of (b) β-catenin, (c) FN and (d) α-SMA in human lung fibroblasts with or without TGF-β challenge. Data are expressed as means ± SEM of three independent experiments. ^*^P<0.05 vs. cells without TGF-β challenge.

TGF-β is a key factor for fibroblast differentiation, and plays critical roles in fibroblast differentiation. To determine whether the expression of β-catenin is correlated with TGF-β-induced activation and differentiation of lung fibroblasts, we assessed the expression of β-catenin in human lung fibroblasts challenged by TGF-β. Western blot analysis revealed that TGF-β challenge (5 ng/ml, 48 h) markedly increased the expression of β-catenin and fibroblast differentiation (Fig. 3a and d).

### TGF-β induces β-catenin expression through NF-κB

**Figure 4:**
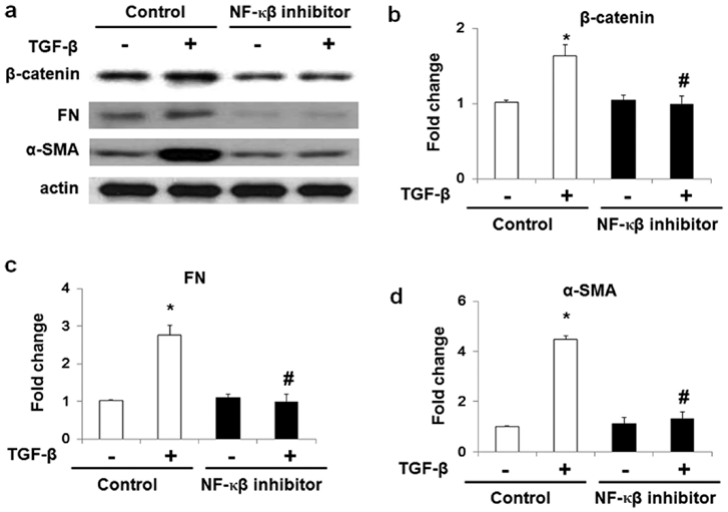
NF-κB inhibitor attenuates TGF-β-induced expression of β-catenin, FN and α-SMA in human lung fibroblasts. (a) Representative and (b–d) quantification of the expression of (b) β-catenin, (c) FN and (d) α-SMA in human lung fibroblasts with or without TGF-β challenge. Data are expressed as means ± SEM of three independent experiments. ^*^P<0.05 vs. cells without TGF-β challenge. ^#^P<0.05 vs. cells without NF-κB inhibitor treatment and with TGF-β challenge.

To investigate the molecular mechanisms of the TGF-β-induced expression of β-catenin expression in lung fibroblasts, we pretreated the WI-38 human lung fibroblast cell line with NF-κB inhibitor (10 μM) for 1 h prior to the TGF-β challenge. As shown in Fig. 4, TGF-β upregulated β-catenin, α-SMA and FN expression in human lung fibroblasts, whereas pretreatment of the NF-κB inhibitor markedly blocked the effects of TGF-β. These data showed that NF-κB signaling pathways were involved in TGF-β-induced β-catenin expression in human lung fibroblasts.

### LPA-induced differentiation of fibroblasts and expression of β-catenin is attenuated by anti-TGF-β antibody

**Figure 5:**
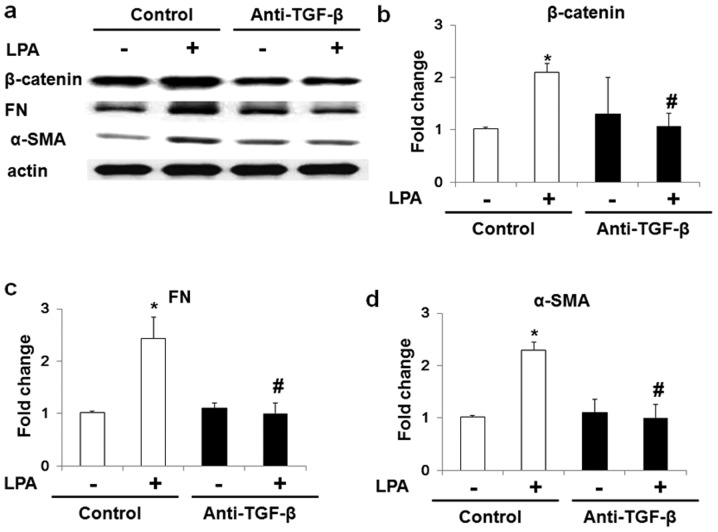
Lysophosphatidic acid (LPA) induced the differentiation and expression of β-catenin in human lung fibroblasts. (a) Representative western blot (b–d) quantification of the expression of (b) β-catenin, (c) FN and (d) α-SMA in LPA-challenged WI-38 cells. Data are expressed as means ± SEM of three independent experiments. ^*^P<0.05 vs. cells with control antibody but without LPA treatment. ^#^P<0.05 vs. LPA-challenged cells with pretreatment of control antibody.

The LPA effects on the differentiation and expression of β-catenin in human lung fibroblasts were examined. As shown in Fig. 5, LPA treatment induced fibroblast differentiation, and increased β-catenin expression in human lung fibroblast. Additionally, to block the effect of secreted TGF-β under LPA challenge, the cells were pretreated with an anti-TGF-β antibody. Pretreatment of anti-TGF-β antibody markedly inhibited the LPA-induced differentiation of human lung fibroblasts and significantly blocked the LPA-induced expression of β-catenin (Fig. 5). These results suggested that LPA also induced the expression of β-catenin in human lung fibroblasts, and this effect at least, partly through LPA induced the expression and secretion of TGF-β.

## Discussion

Idiopathic pulmonary fibrosis (IPF) is a fatal lung disease with no effective pharmacological treatment. Findings of previous studies have shown that following the progression of pulmonary fibrosis, various lipid ligands, cytokines and chemokines were altered (2,4,7,25). TGF-β has also been identified as a key factor for pulmonary fibrogenesis and differentiation in lung fibroblasts (2). Previous studies have proven crosstalk of the TGF-β and Wnt/β-catenin pathways during fibrogenesis (26). However, the role of TGF-β in the expression of β-catenin in fibroblasts remains to be investigated.

As an important component of Wnt/β-catenin pathway, β-catenin is involved in the regulation of branching morphogenesis, regional specialization of the epithelium and mesenchyme and establishment of progenitor cell pools. During pulmonary fibrogenesis, the Wnt/β-catenin signaling pathway contributes to proliferation, differentiation and migration in lung fibroblasts and EMT in lung epithelium cells. Immunohistochemical staining of lung tissue from IPF patients indicated that nuclear β-catenin accumulation was demonstrated in fibroblast foci. Aberrant activation of Wnt/β-catenin signaling and expression of the downstream genes were observed in bronchiolar lesions from IPF patients (27). Expression of β-catenin in nuclear also increased in fibrotic lesions and promoted the migration and proliferation in lung tissue from systemic sclerosis pulmonary fibrosis patients (12). Additional investigation by qPCR demonstrated that the Wnt/β-catenin pathway is expressed and operative in adult lung epithelium (10). In ventilator-induced pulmonary fibrosis, Wnt/β-catenin signaling was activated at an early stage by mechanical ventilation in lungs suggesting that modulation of the Wnt/β-catenin pathway may be a therapeutic option for the prevention of ventilator-induced pulmonary fibrosis (28). In bleomycin-induced pulmonary fibrosis, blockade of the Wnt/β-catenin pathway by β-catenin siRNA or ICG-001, a small molecule that specifically inhibits T-cell factor/β-catenin transcription in a cyclic AMP response-element binding protein binding protein (CBP)-dependent manner, inhibited bleomycin-induced pulmonary fibrosis in mouse (29,30). In lung fibroblasts from IPF patients, the lower expression of α_2_β_1_ integrin resulted in the activation of β-catenin pathway is critical for the proliferation of fibroblasts on collagen expression (20). The abovementioned studies suggested that targeting the expression and activation of β-catenin is a potential therapeutic strategy for IPF.

Recently, S1P and LPA, as well as other lipid ligands, were also proven to be involved in inflammatory reactions (4,9,31–35) and pulmonary fibrosis (4,9). In lung tissue from IPF patients, the level of S1P and LPA were markedly higher than that from control patients (4,7,36). *In vitro* studies also showed that S1P and LPA activated the recruitment, activation and differentiation of lung fibroblasts through their G protein-coupled receptors (4,7,9). LPA has also been found to induce the expression of TGF-β in human lung fibroblasts through the activation of LPA receptor type 2 in lung fibroblasts (9). The present study suggests that LPA induced expression of β-catenin in human lung fibroblasts at least partly through TGF-β.

In conclusion, results of the present study have shown that β-catenin expression increased in lung fibrotic foci and its expression was positively correlated with the activation of lung fibroblasts under pathological conditions *in vitro* and *in vivo*. Additionally, TGF-β increased β-catenin expression through the NF-κB signaling pathway.
